# Web-based interventions to decrease alcohol use in adolescents: a Delphi study about increasing effectiveness and reducing drop-out

**DOI:** 10.1186/s12889-015-1639-z

**Published:** 2015-04-09

**Authors:** Astrid Jander, Rik Crutzen, Liesbeth Mercken, Hein De Vries

**Affiliations:** Department of Health Promotion, Maastricht University, School for Public Health and Primary Care CAPHRI, Maastricht, 6200 MD The Netherlands

**Keywords:** Delphi study, Alcohol use, Adolescents, Web-based interventions, e-Health studies, Computer-tailored (CT) interventions

## Abstract

**Background:**

Web-based computer-tailored (CT) interventions have a high potential to reach a large number of people and effectively change health risk behaviors and their determinants. However, effect studies show small and variable effect sizes, and these interventions also suffer from high drop-out. In this study we explored how Web-based CT interventions can be used effectively to reduce binge drinking in 16- to 18-year-old adolescents.

**Method:**

A three-round Delphi study was conducted. We invited experts to identify strategies to be used in Web-based CT interventions that can effectively decrease binge drinking in adolescents and to rate these strategies by importance. We asked to discriminate between interventions targeted for adolescents and those targeted for parents. Furthermore, we asked experts to suggest strategies for reducing drop-out and to indicate their importance.

**Results:**

Important strategies mentioned by the experts were: encouraging parents to set appropriate rules, encouraging consistent communication, and training refusal skills among adolescents. Concerning the reduction of drop-out from Web-based CT interventions experts came up with suggestions involving the content of the intervention (e.g., relevant material, use of language, tailored messages) but also involving the use of reminders and incentives.

**Conclusions:**

The results of this explorative study provide useful strategies to increase effectiveness and decrease drop-out in future interventions.

**Electronic supplementary material:**

The online version of this article (doi:10.1186/s12889-015-1639-z) contains supplementary material, which is available to authorized users.

## Background

Binge drinking, particularly for adolescents, is associated with a variety of negative consequences, such as fighting, being injured and injuring others [1], dating violence, attempting suicide, smoking, and using other (illicit) drugs [2]. Furthermore, binge drinking (drinking ≥4/5 glasses per occasion for girls/boys) [3] is associated with brain damage and neurocognitive deficits [4] and can impair learning and school performance [2], and causes high societal costs, like health-care and law-enforcement costs, as well as costs for property damage and social work services [5]. Binge drinking is prevalent in Europe with an average of 39% of adolescents having at least one binge drinking occasion in the previous 30 days [6]. Therefore, interventions are needed to reduce binge drinking among adolescents [1,2], which should be targeted at their personal determinants (e.g., socio-cognitive variables) as well as their environment (e.g., parenting and peer influences) [7]. The 16- to 18-year-old adolescent group has been largely understudied, with only a few studies focusing on this age group [8,9]. Most studies focused on either younger adolescents [10-14] or young adults who are 18 years and older [15-17].

An effective way to reduce binge drinking in adolescents could be through Web-based CT interventions. Web-based interventions have the potential to reach a large number of people, as access to the Internet is growing worldwide [18]. Most often these interventions use the Social Cognitive Model (SCT) [19], Transtheoretical Model (TTM) [20], or Theory of Reasoned Action/Planned Behavior (TPB) [21,22] and its determinants to develop the intervention [23]. Interventions built on TPB, however, led to substantially larger effects compared to the other theories [23].

The CT messages are developed by analyses of cognitive determinants of behavior and formulating feedback messages tailored to these determinants [24]. Furthermore, individual characteristics of a person can be taken into account (i.e., demographics), which results in relevant and highly individualized information that is more likely to attract attention [25]. Personalization and feedback have been shown to be effective working mechanisms of CT interventions [26]. CT interventions have been proven to be efficacious in changing health risk behaviors and their determinants [27,28], but effect sizes, although statistically significant, are often only small to medium [27]. This raises the question whether the right strategies were used to target the health behavior and this specific group. There are methods available to target determinants at several levels [29], but relatively little is known regarding how to translate these methods into strategies incorporated into Web-based CT interventions. A review has shown that the most commonly used behavior change techniques included providing information about consequences of behavior, prompting self-monitoring, identifying barriers, and providing problem solving skills, but those associated with the largest effects on behavior in Web-based interventions were stress management and general communication skills training [23]. Other effective strategies were modeling, relapse prevention/coping planning, facilitating social comparison, goal setting, action planning, and feedback on performance [23]. Furthermore, Web-based CT interventions can reach many people, but tend to have high drop-out rates [30-32]. This problem is common in eHealth effect studies and results in less power to reveal potential effects [33]. Some studies investigated the effects of invitations to and incentives of surveys to reduce drop-out rates [34-36] and suggest that using incentives, short questionnaires and personalization of the invitation might be effective in increasing response rates. Yet, there is scarce knowledge about how to design an intervention to target that problem. Therefore, this issue should be addressed when developing Web-based CT interventions.

Although previous research has clearly identified determinants of adolescent binge drinking [16,37,38], to change these determinants some methods are more or less suitable depending on the target group and the way the intervention is delivered [29]. Therefore, this study has two goals: first, we aim to identify the most suitable strategies for Web-based interventions aimed to change determinants and to reduce binge drinking among 16- to 18-year-old adolescents; strategies that may target adolescents’ personal factors, as they have been found to be important determinants of binge drinking [12,39,40], as well as their parents, who still have considerable influence on their children’s alcohol intake during this age period [14,41,42]. Second, we aim to identify strategies that can reduce drop-out of adolescents and parents in Web-based CT interventions.

## Methods

**Figure 1:**
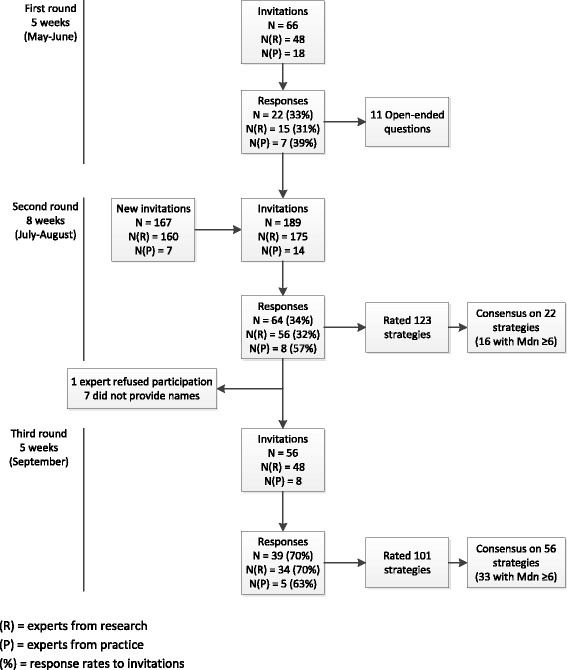
**Overview of the process of the Delphi rounds.**

We conducted a three-round Delphi study during a five-month period (Figure 1). A Delphi study is a method used to structure a group communication process in order to reach consensus to a complex problem [43]. Although the number of rounds required is disputed, it appears that the majority of studies prefer either two or three rounds [43,44]. A three-round method is advantageous, since factors for which no clear consensus has been reached in the second round are offered another time to respondents for a critical review concerning their importance. During each round experts were invited to respond to a specific set of questions. The rounds were iterative in nature and each round took about 10 to 15 minutes to complete. Experts received an e-mail inviting them to participate in an Internet Delphi study. The e-mail contained a link to the online questionnaire. Two weeks after the first invitation a reminder was sent to non-responders, followed by a second reminder if needed after three weeks. Invited experts came from both research and practice backgrounds, to get a broad overview of the existing knowledge from both fields. These experts had experience with alcohol prevention projects or projects to reduce alcohol use for adolescents and young adults. They were invited to indicate whether their expertise involved interventions and studies about binge drinking adolescents targeting adolescents, those targeting parents, or both (Table 1).

**Table 1:** **Field of expertise indicated by the experts**

**Field of expertise**	**First round**	**Second round**	**Third round**
Interventions and studies about binge drinking adolescents targeting adolescents	28%	45%	41%
Interventions and studies about binge drinking adolescents targeting parents	5%	3%	3%
Both	67%	52%	56%

In order to facilitate examination of the data and analyses and support accurate replication attempts to contribute to future meta-analyses [45,46], all study materials (i.e., questionnaires, data, syntax, and output of the analyses) are available at www.sciencerep.org/14.

### First round

The first round consisted of open-ended questions. Four main topics were covered with two to three sub-questions per topic. In order to prompt the experts to think of successful strategies, we first asked for all possible factors that determined binge drinking in adolescents. This technique, called elicitation, is often used to identify salient beliefs underlying behavioral determinants [47] (a list of the identified determinants can be found in the tables in the Additional file 1). The first three topics were about parenting practices/styles/actions, environmental factors, and motivational factors that influence binge drinking and had an identical structure. We then asked the following question: “What are, according to your expertise, effective parenting practices/styles/actions to reduce binge drinking in 16- to 18-year-old adolescents?” Subsequently, we asked: “How would you translate your knowledge about parenting practices/styles/actions into strategies to be used in a Web-based intervention aimed at parents to reduce adolescents’ binge drinking?” If participants indicated that they had experience with interventions targeting parents to reduce adolescents’ alcohol use, they were asked: “According to your expertise, changing which factors have been shown to be especially effective in an intervention aimed at parents to reduce alcohol consumption in adolescents?” The last topic was related to factors to reduce drop-out in interventions targeting adolescents and interventions targeting parents.

We invited 66 international experts to answer the questions from the first round of the Delphi study. Experts with research backgrounds were identified through a search using Google Scholar, PsycINFO and PubMed. If researchers previously published at least three articles on topics that we considered relevant (alcohol, prevention, adolescents, parents, or interventions), they were considered experts in this field. We then conducted a Google search to obtain further information about them on their institute Web sites (e.g., field of expertise, e-mail address) and invited them to participate. We also invited experts with practical backgrounds because they are often actively involved in implementing and conducting interventions, and have thus more experience in the application of interventions in the field. We reached them by approaching established national institutes that are very active in preventing alcohol and drug use (e.g., Trimbos Institute and Mondriaan Verslavingzorg). Eventually, 22 experts from six countries (Australia, Iceland, Sweden, The Netherlands, the United Kingdom, the United States) (33% response rate) filled out the questionnaire (Figure 1).

All answers given by the experts in the first round were categorized into a list of factors and strategies. First, all answers to one question were listed. Second, all double items were deleted. Finally, semantically similar items were taken together. The first step was done by one researcher only. Two more researchers were involved in the second and third steps. Consensus on the final list of items was reached through discussion [48]. The entire research team approved the final questionnaire.

### Second round

All experts from the first round were invited to participate in the second round. In addition, we searched Google Scholar, PsycINFO and PubMed to identify more experts in the relevant fields, as well as abstract books from relevant conferences (e.g., European Health Psychology Society (EHPS) and Kettil Bruun Society (KBS)). Of the 189 identified and invited experts, 64 from 11 countries (Australia, Brazil, Canada, Germany, Norway, Portugal, Sweden, Switzerland, The Netherlands, the United Kingdom, and the United States) responded to our request to participate in the second round (Figure 1).

Because we were mainly interested in the strategies to change determinants, we only asked for determinants in the first round to elicit the eligible strategy.

Experts from the second round were presented a list with all strategies to reduce binge drinking and strategies to reduce drop-out that were identified during the first round and to indicate the importance of each strategy using a seven-point Likert scale ranging from 1 (not important at all) to 7 (extremely important).

The data were analyzed by calculating the median score (Mdn), to indicate the importance of every strategy, and the interquartile deviations (IQD), to get an impression of the degree of consensus of the experts on the strategy [49]. The median score can be defined as the score that falls exactly in the middle of a group of scores, meaning that exactly one half of all obtained scores lies above and the other half of all scores lies below this median score. In this study a median score of ≥6 is considered important. The IQD is a measure used to express the degree of consensus obtained, with a higher IQD referring to a smaller degree of consensus. When using a seven-point scale, IQDs with a value of ≤1 (more than 50% of the opinions fall within one point of the scale) indicate good consensus [43].

### Third round

All experts that participated in the second round were invited to take part in the third and final round of the Delphi study. One expert refused participation in the final round and seven experts did not provide their names in the questionnaire. We therefore invited 56 experts in the final round, 39 of whom completed the questionnaire (Figure 1).

The questionnaire, including the feedback about median and IQD for each item from the second round, was sent to the participants to re-rate their answers from the prior round. Of all items, 17.9% had an IQD ≤ 1 and were taken out of the questionnaire. This resulted in the third round questionnaire consisting of 101 questions.

### Ethics approval

Ethical approval of the Regional Medical Ethics committee in the Netherlands was not necessary because participants in this study were not “subjected to procedures or required to follow certain rules of behavior” (http://www.ccmo.nl/en/your-research-does-it-fall-under-the-wmo).

## Results

During the first round a number of determinants of adolescent binge drinking were identified. For these determinants, the experts defined strategies to change that determinant in order to reduce binge drinking. These strategies, including the results of the second and third rounds, are listed in Tables 2, 3, 4 and 5.

**Table 2:** **Results for items related to effectiveness of strategies to reduce binge drinking in 16- to 18-year-old adolescents in an intervention targeting parents**

**Strategy**	**Second round**		**Third round**	
	**N = 64**		**N = 39**	
	**Mdn**	**IQD**	**Mdn**	**IQD**
1. Advise parents not to provide adolescent child with alcohol	6	2	6	2
2. Provide normative information (e.g., actual figures) to parents about adolescent drinking	5	1.5	5	1
3. *Advise parents to have clear and consistent rules	**7**	**1**	-	-
4. Give parents the opportunity to communicate with other parents to have the same kind of rules	5	2	5	2
5. Present different parenting styles and its relation with drinking and other variables that relate to positive youth development	5	1.25	5	2
6. *Provide approaches to communication (particularly conflict resolution)	**6**	**1**	-	-
7. *Demonstrate an authoritative parenting style as opposed to authoritarian and permissive parenting styles	6	2	**6**	**1**
8. Present evidence regarding the efficacy of the authoritative approach in a way that is palatable for parents	5	2	5	2
9. Describe ways of using authoritative parenting styles	6	2	6	1.50
10. Encourage parents to spent time with their adolescents	6	2	6	1.50
11. Advise parents to talk to their adolescent children regularly about things that interest the adolescent	6	1.75	6	2
12. *Provide parents with evidence that delaying introduction to alcohol consumption helps protect their adolescent children from alcohol-related harms	6	2	**6**	**1**
13. *Give immediate and tailored feedback to the parents	6	1.25	**6**	**1**
14. *Demonstrate more or less effective communication styles	**6**	**1**	-	-
15. Build communities on special topics (celebration of 16th birthday)	5	2	4	2
16. Educate parents about negative consequences for the development of the brains until age 24	5	2	5	2
17. Emphasize short term negative effects of alcohol on adolescents	5	2	5	2
18. *Advise parents to get to know the whereabouts of the adolescent	6	2	**6**	**1**
19. *Advise parents to get to know the friends of the adolescent	6	2	**6**	**1**
20. Advise parents to conduct family bounding activities (e.g., having evening meal together)	6	2	6	2
21. Make clear to parents that their own youth habits differ from the current youth habits	5	1.75	5	2
22. Advise parents to have clear expectations towards the adolescent not to drink alcohol	6	2	6	1.25
23. Advise parents to communicate about expectations not to drink alcohol towards the adolescent	6	2	6	1.25
24. Emphasize that communication between parent and child has to be firm	5	2	5	2
25. *Emphasize that communication between parent and child has to be consistent	**7**	**1**	-	-
26. *Emphasize that communication between parent and child has to be kind	**6**	**1**	-	-
27. *Emphasize that communication between parent and child has to be open	**6**	**1**	-	-
28. Emphasize that communication between parent and child has to be healthy	6	2	6	2
29. *Emphasize that communication between parent and child has to be from positive quality	6	2	**6**	**1**
30. Advise parents to come to agreements with their adolescent child regarding alcohol consumption	5	2	5	1.50
31. Advise parents to be a good role model (do not drink (much) in presence of the adolescent)	6	2	6	1.25
32. Advise parents to monitor the alcohol consumption of their adolescent child	5	1	-	-
33. Advise parents not to serve alcohol at home	4	3	4	2
34. *Emphasize the importance of responsive parenting (parents who expect a lot from their adolescent child and provide them with a sense of self-efficacy)	**6**	**1**	-	-
35. *Consistent adolescent management practices (balancing the two dimensions of ‘care’ and ‘control’) regarding alcohol consumption	6	2	**6**	**0.75**
36. *Advise parents to have active interest in the adolescents life	6	2	**6**	**1**
37. Advise parents to conduct activities that the adolescent enjoys	5	1.75	5	1
38. *Advise parents to instruct older siblings not to provide their younger siblings with alcohol	**6**	**1**	-	-
39. *Emphasize that family can continue to be a moderating influence throughout adolescence and even young adulthood because parents usually affect long term goals and values	6	2	**6**	**1**
40. *Strengthen parents self-efficacy towards making agreements and setting rules	6	2	**6**	**1**

*Mdn: median scores.*

*IQD: interquartile deviation.*

*-: these items had an IQD ≤ 1 in the second round and did not reappear in the third round.*

*Strategies that are in italics and marked with an asterisk were identified as important (Mdn ≥ 6) and experts had reached consensus on (IQD ≤ 1).*

In the first round, 70 factors that determine binge drinking in adolescents, 40 strategies targeting parents, and 47 strategies targeting adolescents were identified. With regard to reducing drop-out, 17 and 19 strategies were identified for adolescents and parents, respectively. In the second round, experts reached consensus (IQD ≤ 1) on 16 strategy items that were considered important (Mdn ≥ 6). In the third round, experts reached consensus on 33 important items. Both rounds taken together delivered a consensus of 49 out of 123 items (39%).

### Strategies targeting parents

Concerning strategies for interventions that target parents, experts agreed on eight important items during the second round and another 10 items during the third round (Table 2). The strategies considered relevant can be categorized into the following: 1) parenting practices like setting rules, communication about alcohol, and monitoring of the child, and 2) parenting styles, such as being responsive and interested in the child.

### Strategies targeting adolescents

A further goal of this study was to identify strategies to reduce binge drinking in adolescents in an intervention aimed at adolescents; these results are depicted in Table 3. Experts agreed on three important strategies during the second round and on another four during the third round. Most prominent were strategies to increase refusal skills. Other strategies were related to coping with negative emotions, dealing with drinking in social situations, and developing decision-making skills.

**Table 3:** **Results for items related to effectiveness of strategies to reduce binge drinking in 16- to 18-year-old adolescents in an intervention targeting adolescents**

**Strategy**	**Second round**		**Third round**	
	**N = 61**		**N = 35**	
	**Mdn**	**IQD**	**Mdn**	**IQD**
1. Present dramatic portrayals that adolescents can identify with	4	2.75	4	1.50
2. Provide normative data regarding peer drinking	5	2	5	2
3. Lessen the “coolness” factor of drinking: use role models that are cool without alcohol	5	2	5	2
4. Place an emphasis on how adolescents make meaning of their own drinking and how that relates to their own drinking (e.g., “this happens to others but not to me”)	5	2	5	1
5. Adolescents should be reminded that the choice to drink is theirs and theirs alone	5	2	5	1.25
6. Explain why choosing not to drink is a good choice	5	1.75	5	1
7. Add or remove alcohol cues in a pictorial scenario to demonstrate how social environmental cues can manipulate alcohol consumption	5	2	5	1
8. Use prevalence overestimates reduction (present their own use, their perception of peer use and actual peer use of every 100 peers)	5	2	5	2
9. Role playing games creating your own avatar	4.50	3	4	2
10. Present social situations and ask them how they would react and present the different (positive and negative) consequences	5	1	-	-
11. *Provide the opportunity to try out different reactions and their consequences in social situations	**6**	**1**	-	-
12. Show a movie with victims that have been significantly affected by drinking (for example road accidents)	3	2	2	1
13. Improving skills in dealing with general life issues	6	3	6	2
14. Encouraging adolescents’ interests in other activities that do not involve alcohol consumption	5	2	5	1
15. Increase knowledge about detrimental effects of alcohol before the age of 24 years	4	2.75	5	1.25
16. *Increasing refusal skills (ability to say “no”)	**6**	**1**	-	-
17. *Increase self-efficacy over their ability to refuse to engage in binge drinking	6	2	**6**	**0**
18. *Increase their levels of perceived control whether or not they could refuse to engage in binge drinking	6	1.25	**6**	**0**
19. *Train self-control (the ability to set limits for oneself)	6	2	**6**	**0.50**
20. Provide knowledge about the harm of binge drinking / negative consequences of alcohol	5	2	5	1
21. Increasing the sense of risk through emphasizing the short term consequences	5	1.50	5	1
22. Provide accurate information about alcohol expectancies	5	2	5	1
23. *Provide ways to cope with negative mood states other than drinking	**6**	**1**	-	-
24. Stress that there are alternatives to alcohol and binge drinking	5	2	5	1
25. Focus on how adolescents make meaning of their own drinking (arguments that adolescents use to defend their alcohol consumption)	5	2	5	1
26. Show good graphic vomit shots	1	2	1	0
27. Show them embarrassing behavior due to binge drinking	2	3.75	2	2
28. Develop planning and communication skills	5	2	5	1
29. *Develop decision making skills	5	1.25	**6**	**1**
30. Giving advice to others on the topic	4	2.50	3.50	1
31. Emphasize the benefits of positive choices	5	2	5	1.25
32. Check out their personality and tailor the intervention on this personality: fearful personality	4	3	4	2
33. Check out their personality and tailor the intervention on this personality: having negative thinking patterns	5	3	4.50	1.75
34. Check out their personality and tailor the intervention on this personality: sensation seeking personality	6	2	6	2
35. Check out their personality and tailor the intervention on this personality: impulsive personality	6	2	5.50	2
36. Discriminate motives to drink and tailor intervention on these: drinking to deal with negative emotions (coping motives)	6	2	5	2
37. Discriminate motives to drink and tailor intervention on these: drinking to enhance positive emotions (enhancement motives)	6	2	5.50	2
38. Discriminate motives to drink and tailor intervention on these: drinking to be social (social motives)	6	2	5.50	2
39. Discriminate motives to drink and tailor intervention on these: drinking to conform to the group (conformity motive)	6	2	5	2
40. Changing adolescents’ positive attitude towards binge drinking	5	2	5	1
41. Creating awareness of ambivalence (balance between positive and negative consequences of drinking)	5	2	5	2
42. Strengthening those aspects that are already seen by the adolescent as positive consequences of not drinking	5	1	-	-
43. Strengthening those aspects that are already seen by the adolescent as negative consequences of drinking	5	1	-	-
44. Emphasize the possibility of getting high status by acting healthy	5	3	5	1.25
45. Stimulate action planning skills on preventing binge drinking	5	2	5	1
46. Stimulate to plan moderate drinking beforehand (e.g., special events or holiday)	5	2	5	1
47. Encouraging the adolescents’ sense of autonomy and self esteem	5	2	5	1

*Mdn: median scores.*

*IQD: interquartile deviation.*

*-: these items had an IQD ≤ 1 in the second round and did not reappear in the third round.*

*Strategies that are in italics and marked with an asterisk were identified as important (Mdn ≥ 6) and experts had reached consensus on (IQD ≤ 1).*

### Strategies to reduce drop-out of adolescents

Concerning drop-out of adolescents, experts agreed on one important strategy during the second round and another 12 strategies in the third round. During the second round, experts only agreed on the importance of incentives. During the third round, strategies related to design and content of the intervention and the importance of reminders were emphasized (Table 4).

**Table 4:** **Results for items related to importance of several factors to reduce drop out of adolescents in a Web-based intervention to reduce binge drinking in 16- to 18-year-old adolescents**

**Strategy**	**Second round**		**Third round**	
	**N = 56**		**N = 34**	
	**Mdn**	**IQD**	**Mdn**	**IQD**
1. *Monetary incentives	6	2.25	**6**	**0.50**
2. *Non-monetary incentives (e.g., movie tickets)	**6**	**1**	-	-
3. *Reminder per e-mail	6	2.75	**6**	**0.25**
4. *Reminder per sms (text message)	6	2	**6**	**0.25**
5. *Engaging graphics	6	3	**6**	**1**
6. *Self-assessment with personalized feedback	6	2	**6**	**1**
7. *Use of highly relevant material	6	2	**6**	**0**
8. *Attractive design	6	2	**6**	**0.25**
9. *Inspiring topics	6	2	**6**	**0.25**
10. *Using language that relates to the adolescents	6	2	**6**	**1**
11. *Use as little text as you can get away with	6	2	**6**	**0.50**
12. *Use as much interaction as possible	6	2	**6**	**0.50**
13. Use of humor	5	3	5	1.50
14. *Engrossing website	6	1.75	**6**	**0**
15. The use of the website should be addictive itself	4	3	4	1.75
16. Give points to earn (e.g., game component)	5	2	5	2
17. Set little goals to achieve during the intervention	5	1	-	-

*Mdn: median scores.*

*IQD: interquartile deviation.*

*-: these items had an IQD ≤ 1 in the second round and did not reappear in the third round.*

*Strategies that are in italics and marked with an asterisk were identified as important (Mdn ≥ 6) and experts had reached consensus on (IQD ≤ 1).*

### Strategies to reduce drop-out of parents

Concerning drop-out of parents, experts agreed on four important strategies in the second round and seven in the third round. Here the important strategies were related to the content and design of the intervention (e.g., usability, feasible recommendations, tailoring the intervention) and the use of reminders (Table 5).

**Table 5:** **Results for items related to importance of several factors to reduce drop out of parents in a Web-based intervention to reduce binge drinking in 16- to 18-year-old adolescents**

**Strategy**	**Second round**		**Third round**	
	**N = 56**		**N = 34**	
	**Mdn**	**IQD**	**Mdn**	**IQD**
1. Monetary incentives	5	3	5	1.75
2. Non-monetary incentives (e.g., movie tickets)	5	2	5	1
3. *Reminders per e-mail	6	2	**6**	**1**
4. *Reminders per sms (text message)	6	2.50	**6**	**0.75**
5. *Use of highly relevant material	6	2	**6**	**0.50**
6. *Interesting topics	6	2	**6**	**0**
7. *Ensuring that they realize that doing this will make a difference	**6.5**	**1**	-	-
8. *Use of language that does not sound pompous or may be interpreted as condescending	6	2	**6**	**1**
9. *Make clear that it is understood that parents are the best experts when it comes to their children and that parents want what’s best for their children and that being a parent can be extremely difficult	6	2	**6**	**0**
10. *Recommendations need to be realistic and feasible	**6.5**	**1**	-	-
11. Engrossing website	5	2	5	0.25
12. The use of the website should be addictive itself	3	3.50	3	2
13. Compelling set of lessons	5	2	5	1
14. *Make the need for the intervention salient to parents	6	2	**6**	**0**
15. Attractive design	5	1	-	-
16. *Usability	**7**	**1**	-	-
17. *Tailored	**6**	**1**	-	-
18. Tips / reaction from an expert	5	2	5	1
19. Parents should have the possibility to communicate with each other (e.g., forum)	5	2	5	1.50

Mdn: median scores.

IQD: interquartile deviation.

-: these items had an IQD ≤ 1 in the second round and did not reappear in the third round.

Strategies that are in italics and marked with an asterisk were identified as important (Mdn ≥ 6) and experts had reached consensus on (IQD ≤ 1).

## Discussion

The aim of this Delphi expert study was to gather expertise on effective strategies to be used in Web-based CT interventions to reduce binge drinking in 16- to 18-year-old adolescents. Some of the important strategies that could be used in an intervention targeted at parents are already described in the literature like specific parenting practices such as monitoring the adolescents’ whereabouts and friends [50-52], and being a responsive and interested parent [53]. Results from previous studies regarding communication about alcohol, which was also considered important by our experts, are more heterogeneous. Some studies found a positive effect of communication on alcohol consumption [41,42], others show no effect of communication or even detrimental effects [54]. Our experts highlighted specific aspects of communication other than just frequency. They recommend that communication should be consistent, kind, open, and of positive quality. One study [42] found indeed a difference in quality and frequency of communication about alcohol use, with quality being negatively associated with alcohol use and frequency being positively associated. To obtain more insight about which aspects of communication are useful in this context, more research is recommended [14]. Nevertheless, when advising parents about communication with their children about alcohol, attention should be paid to communication being of good quality, open, kind, and consistent, as indicated by the experts in this study, rather than very frequent. Furthermore, experts in this study placed a high importance on setting clear and consistent rules which is in accordance with previous research [14,42,55]. However, what kinds of rules (e.g., zero tolerance rules, rules that are in line with the health guidelines, rules that are self-set by parents) have a different effect in reducing alcohol use in a target group that is legally allowed to purchase alcohol in comparison with other countries where this is illegal is still unclear. To our knowledge, no research has yet been conducted to entangle this problem. Finally, our experts reached consensus on the importance of emphasizing that the family continues to have influence throughout adolescence and young adulthood and that parents’ self-efficacy toward making agreements and setting rules should be strengthened. Applying these recommendations in Web-based interventions could mean creating a Web site for parents where they can obtain information about the importance of setting rules and communicating with their adolescent about alcohol and how to do this. To make this information as personalized and relevant as possible, computer-tailoring strategies could be used, thus assessing current communication and rule setting with the help of questionnaires and then providing the parents with personalized feedback based on their answers. These computer-tailored feedback messages could be either text-based or video-based, as both have been proven to be effective; however, video messages are preferred as they have been shown to be slightly more effective compared to text-based messages [56].

Regarding effective strategies targeting adolescents to reduce binge drinking, experts agreed on the importance of giving adolescents the opportunity to try out different reactions and their consequences in social situations, increasing refusal skills and perceived control in adolescents, and providing opportunities to cope with negative emotions in other ways than drinking. The Social Cognitive Theory (SCT) [57] assumes that self-efficacy is a very important factor that influences whether people perform a specific behavior. Self-efficacy can be increased by, for example, enactive mastery experience, modeling, or verbal persuasion [29]. In enactive mastery experience individuals are confronted with different situations that increase in difficulty. They try to master them and receive feedback on their performance. One possibility is to confront adolescents with social situations in which alcohol is available and increase the difficulty by adding people and pressure to drink alcohol to the situations. This could be simulated in a Web-based intervention by using animations or videos that allow users to make choices that lead to different scenarios that result from their choices. Using modeling as a technique in Web-based interventions could be implemented by using short videos that show how other adolescents successfully refuse alcoholic drinks. Verbal persuasion techniques could be implemented by showing videos of adolescents that explain how they refuse drinks and encourage the adolescent that he or she also has the capability of refusing drinks and resisting peer pressure. Interventions that focused on preventing alcohol use in young adolescents (11 to 14 years of age) found that teaching techniques to manage social influences and pressure to drink and offering alternatives to alcohol [58] are effective in reducing alcohol use [59]. Several other studies have shown that making coping plans was predictive for long-term lifestyle change in rehabilitation patients after discharge [60] and increased abstinence rates in quitters from smoking [61]. These studies indicate that coping plans might be helpful in maintaining a healthy lifestyle and preventing unhealthy behavior, like binge drinking, if adolescents formulate coping plans for situations that are difficult for them.

Another goal of this Delphi study was to identify effective strategies to reduce drop-out as this constitutes a major problem to Web-based interventions. Gathering this expertise is very important, as there is little research available on effective strategies to reduce drop-out. For interventions aimed at adolescents, experts reached consensus on different strategies that can be divided into three categories: 1) providing incentives (non-monetary and monetary); 2) creating an appealing content (setting small goals to achieve during the intervention; using engaging graphics; offering self-assessment with personalized feedback; using highly relevant material, attractive designs, and inspiring topics; using language that relates to the adolescents; providing as little text and as much interaction as possible; designing an engrossing Website); and 3) sending reminders (e-mail and text messages). Generally, we can discriminate two kinds of drop-out. The first is intervention drop-out, meaning that participants drop-out during the intervention, and thus are not fully exposed to the intervention content which can negatively affect public health impact of the intervention [33]. The other form is drop-out at follow-up assessment, thus participants not returning to a follow-up assessment. This form of drop-out diminishes the possibility to reveal possible effects [33]. Both forms are problematic to intervention trials. Some of the strategies mentioned by the experts can either be used to reduce both forms of drop-out (e.g., providing incentives for completion of the intervention and for returning to the follow-up assessments), but other strategies work better to reduce one form of drop-out (e.g., using engaging graphics in the intervention to reduce intervention drop-out). The results regarding intervention content, which would be useful to reduce intervention drop-out, are of particular importance as limited experimental research has been conducted to test the effect of the content and layout of an intervention on drop-out rates. Most importantly, when creating a Web-based intervention, developers and researchers should collaborate closely with the target group to ensure that the chosen material is attractive, inspiring, and relevant. Methods to do this could be focus group interviews or a panel of the target group that evaluates all materials and provides feedback. Web-based interventions should be pilot tested and usability tests should be conducted in order to see how the intervention is used and understood and to get immediate feedback regarding which parts are appreciated and which not. Using computer-tailoring strategies will make the intervention much more personalized and relevant; however, in order to use the right language, motivational interviewing (MI) techniques [62] could be more appropriate. Although motivational interviewing usually is provided through personal contact between a professional therapist and a client, motivational interviewing techniques have already been successfully used in Web-based interventions to promote physical activity [63,64]. Through the use of computer tailoring, where responses are tailored to the answers given in the program, a dialog between the program and the user can be simulated [65,66]. One of the recommendations is to use a combination of open-ended and multiple choice questions for the MI questions. Open-ended questions can stimulate simple reflection and enable autonomy support while automated feedback messages to multiple choice questions can stimulate skillful reflections [64]. An experiment with an avatar to strengthen the social relationship with the user was not associated with higher intervention impact [63]. Recently, games for education and health promotion purposes, so-called “serious” games, have been developed and tested. The results concerning knowledge acquisition and attitude and behavior change are promising [67,68]. They further seemed to increase intrinsic motivation in adolescents [69,70], which is an important factor for continued intervention use.

When it comes to reducing drop-out in interventions aimed at parents, similar strategies can be used as experts again came up with many strategies relating to the content of the intervention and emphasized the importance of using reminders (e-mail or text messages). With regard to the content, experts mentioned the following strategies: ensuring parents realize following the intervention would make a difference; providing realistic and feasible recommendations; tailoring the intervention; using highly relevant material; providing interesting topics; using language that does not sound pompous or may be interpreted as condescending; ensuring parents they are the experts when it comes to their children; acknowledging that being a parent can be extremely difficult; and making the intervention salient to the parents. In addition to the earlier suggestions (i.e., collaborate closely with target group, use tailoring or motivational interviewing techniques), parents might also benefit from an approach based on goal-setting theory [71] in order to make realistic and feasible recommendations. This could be designed by creating a tool in which parents can choose from a series of sub-goals (e.g., have a first conversation with my child about alcohol, come to an agreement with my child about the amount of alcohol he/she is allowed to drink, make my rules clear to my child and explain consequences of noncompliance). For every sub-goal, further guidance can be provided regarding how to reach the goal, either text-based or, preferably, video-based [56]. Other research thus far has shown that using a tunneled Web site, where visitors are more guided and have less control, increased the time spent on the Web site, number of pages visited, and knowledge gained compared to a Web site where the visitor could move freely [72]. Furthermore, there is some literature available about methods to increase response to postal and electronic questionnaires. A review [34] of this literature identified some effective strategies: giving non-monetary incentives; offering survey results; using shorter questionnaires; personalizing electronic questionnaires by addressing the participants by name, using a picture and white background on the invitation; using interesting (relevant to participants) questions; sending reminders after the initial invitation; including a statement that others had responded; and setting a response deadline. Providing the incentive together with the questionnaire, rather than after the questionnaire was completed, increased response rates [34]. Despite these effective methods, many methods to increase response rates have not proven to be effective, including monetary incentives for online questionnaires [34], contingent versus unconditional incentives [35], or offering cash lotteries (big and small amounts) as incentive [36]. However, these results relate to survey research and not intervention research. Most of the strategies that have been evaluated on effectiveness are related to invitations or reminders to respond to a questionnaire, or incentives that participants received. More research is needed to test whether attractive, relevant, and interesting content can also reduce drop-out during an intervention. In particular, experimental research is needed, as most studies thus far were based on observational research [34-36], which does not allow conclusions about causal relationships.

Given the vast amount of mentioned strategies by the experts it becomes clear that there are many possibilities to decrease drop-out rates in Web-based interventions, but only few have been proven to be effective. Although it may seem wise to combine several strategies in order to increase their impact on retention rates, more experimental research is also needed to test unique and potential interaction effects of these strategies.

### Limitations and strengths

Finally, we noticed that experts only agreed on a few important strategies to reduce binge drinking in adolescents in interventions targeting adolescents (7 out of 47) compared to interventions targeting parents (18 out of 40). We checked whether this could be an artifact of the sample selection, but researchers of parent-based interventions were not oversampled. Therefore, this finding could indicate that adolescents are a particularly difficult target group and that only a few strategies have proven to be effective. We included experts with research and practice background to get a broad overview of the existing knowledge from both fields. It would be interesting to compare strategies from researchers and practitioners to look for similarities and differences. Unfortunately, our sample of practitioners was too small to make meaningful comparisons, but we would recommend this for future research.

We also want to mention that the response rate from the first two rounds compared to the last round was relatively low (33% and 34% compared to 70%, respectively). Yet, comparable response rates have been reported in other Delphi studies [73,74]. The increase from the second to the third round may indicate that once experts agreed to participate in this study, this was likely to predict continued participation. Furthermore, there is no clear indication about decent panel sizes or acceptable response rates [44], so our goal was to reach saturation of information in the first round. We reached this goal with the answers provided by 22 experts participating in the first round.

## Conclusion

This Delphi study identified strategies that can be used in a Web-based CT intervention to reduce binge drinking in 16- to 18-year-old adolescents and strategies to reduce drop-out rates from these interventions. The results of this explorative study can be used to inform future interventions.

## Additional file

Additional file 1: Table S1. Effective parenting practices/styles/actions. **Table S2.** Social environmental factors. **Table S3.** Motivational factors.

## References

[1] Swahn MH, Simon TR, Hammig BJ, Guerrero JL (2004). Alcohol-consumption behaviors and risk for physical fighting and injuries among adolescent drinkers. Addict Behav.

[2] Miller JW, Naimi TS, Brewer RD, Jones SE (2007). Binge Drinking and Associated Health Risk Behaviors Among High School Students. Pediatrics.

[3] Verdurmen J, Monshouwer K, Dorsselaer S, Lokman S, Vermeulen-Smit E, Vollebergh W (2011). Jeugd en riskant gedrag 2011.

[4] Zeigler DW, Wang CC, Yoast RA, Dickinson BD, McCaffree MA, Robinowitz CB (2005). The neurocognitive effects of alcohol on adolescents and college students. Prev Med.

[5] Rehm J, Mathers C, Popova S, Thavorncharoensap M, Teerawattananon Y, Patra J (2009). Global burden of disease and injury and economic cost attributable to alcohol use and alcohol-use disorders. Lancet.

[6] Hibell B, Guttormsson U, Ahlström S, Balakireva O, Bjarnason T, Kokkevi A, Kraus L: The 2011 ESPAD Report: Substance Use Among Stundents in 36 European Countries. 2011.

[7] Green LW, Kreuter MW (1999). Health Promotion Planning: an educational and environmental approach 3rd edn.

[8] Voogt CV, Kleinjan M, Poelen EA, Lemmers LA, Engels RC (2013). The effectiveness of a web-based brief alcohol intervention in reducing heavy drinking among adolescents aged 15–20 years with a low educational background: a two-arm parallel group cluster randomized controlled trial. BMC Public Health.

[9] Kuntsche E, Knibbe R, Gmel G, Engels R (2006). Who drinks and why? A review of socio-demographic, personality, and contextual issues behind the drinking motives in young people. Addict Behav.

[10] Jones DJ, Hussong AM, Manning J, Sterrett E (2008). Adolescent alcohol use in context: The role of parents and peers among African American and European American youth. Cult Divers Ethn Minor Psychol.

[11] MacPherson L, Magidson JF, Reynolds EK, Kahler CW, Lejuez CW (2010). Changes in sensation seeking and risk-taking propensity predict increases in alcohol use among early adolescents. Alcohol Clin Exp Res.

[12] Marcoux BC, Shope JT (1997). Application of the Theory of Planned Behavior to adolescent use and misuse of alcohol. Health Educ Res.

[13] Marsden J, Boys A, Farrell M, Stillwell G, Hutchings K, Hillebrand J (2005). Personal and social correlates of alcohol consumption among mid-adolescents. Br J Dev Psychol.

[14] Van der Vorst H, Engels RCME, Meeus W, Dekovi M, Van Leeuwe J (2005). The role of alcohol-specific socialization in adolescents’ drinking behaviour. Addiction.

[15] Clapp JD, Shillington AM (2001). Environmental Predictors of Heavy Episodic Drinking. Am J Drug Alcohol Abuse.

[16] Courtney KE, Polich J (2009). Binge Drinking in Young Adults: Data, Definitions, and Determinants. Psychol Bull.

[17] Ham LS, Hope DA (2003). College students and problematic drinking: A review of the literature. Clinical Psychological review.

[18] Bewick BM, Trusler K, Barkham M, Hill AJ, Cahill J, Mulhern B (2008). The effectiveness of web-based interventions designed to decrease alcohol consumption–A systematic review. Preventive Medicine: An International Journal Devoted to Practice and Theory.

[19] Bandura A (1986). Social Foundations of Thought and Action: A Social Cognitive Theory.

[20] Prochaska JO, DiClemente CC (1983). Stages and processes of self-change of smoking: toward an integrative model of change. J Consult Clin Psychol.

[21] Fishbein M (1979). A theory of reasoned action: Some applications and implications. Nebr Symp Motiv.

[22] Ajzen I (1991). The theory of planned behavior. Organ Behav Hum Decis Process.

[23] Webb LT, Joseph J, Yardley L, Michie S (2010). Using the Internet to Promote Health Behavior Change: A Systematic Review and Meta-analysis of the Impact of Theoretical Basis, Use of Behavior Change Techniques, and Mode of Delivery on Efficacy. J Med Internet Res.

[24] Dijkstra A, De Vries H (1999). The development of computer-generated tailored interventions. Patient Educ Couns.

[25] De Vries H, Brug J (1999). Computer-tailored interventions motivating people to adopt health promoting behaviours: Introduction to a new approach. Patient Educ Couns.

[26] Dijkstra A (2005). Working mechanisms of computer-tailored health education: evidence from smoking cessation. Health Educ Res.

[27] Krebs P, Prochaska JO, Rossi JS (2010). A meta-analysis of computer-tailored interventions for health behavior change. Prev Med.

[28] Noar SM, Benac CN, Harris MS (2007). Does tailoring matter? Meta-analytic review of tailored print health behavior change interventions. Psychol Bull.

[29] Bartholomew LK, Parcel GS, Kok G, Gottlieb NH, Fernandez ME (2011). Planning Health Promotion Programs: An Intervention Mapping Approach.

[30] Elfeddali I, Bolman C, Candel M, Wiers R, Vries Hd. Preventing Smoking Relapse via Web-Based Computer Tailored Feedback: A Randomized Controlled Trial. J Med Internet Res 2012, 14(4). 10.2196/jmir.2057 PMC3510689 22903145

[31] De Vries H, Logister M, Krekels G, Klaasse F, Servranckx V, van Osch L. Internet-Based Computer Tailored Feedback on Sunscreen Use J Med Internet Res 2012, 14(2). 10.2196/jmir.1902 PMC3376524 22547528

[32] Kohl LFM, Crutzen R, Vries NK (2013). Online Prevention Aimed at Lifestyle Behaviors: A Systematic Review of Reviews. J Med Internet Res.

[33] Eysenbach G. The Law of Attrition. J Med Internet Res 2005, 7(1). 10.2196/jmir.7.1.e11 PMC1550631 15829473

[34] Edwards PJ, Roberts I, Clarke MJ, DiGuiseppi C, Wentz R, Kwan I, Cooper R, Felix LM, Pratap S. Methods to increase response to postal and electronic questionnaires. Cochrane Database of Systematic Reviews 2009(3):1-527 10.1002/14651858.MR000008.pub4 PMC8941848 19588449

[35] Göritz AS (2005). Contingent versus unconditional incentives in WWW-studies. Metodolosky Zvezki.

[36] Göritz AS (2006). Cash lotteries as incentives in online panels. Soc Sci Comput Rev.

[37] Jander A, Mercken L, Crutzen R, Vries H (2013). Determinants of binge drinking in a permissive environment: focus group interviews with Dutch adolescents and parents. BMC Public Health.

[38] Wilks J, Callan VJ, Derek AA (1989). Parent, Peer and Personal Determinants of Adolescent Drinking. Br J Addict.

[39] Cooper ML (1994). Motivations for alcohol use among adolescents: Development and validation of a four-factor model. Psychol Assess.

[40] Kuntsche E, Knibbe R, Gmel G, Engels R (2005). Why do young people drink? A review of drinking motives. Clin Psychol Rev.

[41] Turrisi R, Jaccard J, Taki R, Dunnam H, Grimes J (2001). Examination of the short-term efficacy of a parent intervention to reduce college student drinking tendencies. Psychol Addict Behav.

[42] Spijkerman R, Van den Eijnden RJJM, Huiberts A (2008). Socioeconomic Differences in Alcohol-Specific Parenting Practices and Adolescents’ Drinking Patterns. Eur Addict Res.

[43] Linstone HA, Turoff M (1975). The Delphi Method: Techniques and Applications.

[44] Mullen P (2003). Delphi: myths and reality. J Health Organ Manag.

[45] Crutzen R, Peters G-JY, Abraham C: What about trialists sharing other study materials? BMJ 2012, 345. 10.1136/bmj.e8352 23229066

[46] Peters G-JY, Abraham C, Crutzen R (2012). Full disclosure: doing behavioural science necessitates sharing. The European Health Psychologist.

[47] Fishbein M, Ajzen I (2010). Predicting And Changing Behavior The Reasoned Action Approach.

[48] Crutzen R, De Nooijer J, Brouwer W, Oenema A, Brug J, De Vries NK (2008). Internet-delivered interventions aimed at adolescents: a Delphi study on dissemination and exposure. Health Educ Res.

[49] Jones J, Hunter D (1995). Consensus methods for medical and health services research. Br Med J.

[50] Kim Y-M, Neff JA (2010). Direct and indirect effects of parental influence upon adolescent alcohol use: A structural equation modeling analysis. Journal of Child & Adolescent Substance Abuse.

[51] Beck KH, Boyle JR, Boekeloo BO (2003). Parental monitoring and adolescent alcohol risk in a clinic population. Am J Health Behav.

[52] Borawski EA, Ievers-Landis CE, Lovegreen LD, Trapl ES (2003). Parental monitoring, negotiated unsupervised time, and parental trust: The role of perceived parenting practices in adolescent health risk behaviors. J Adolesc Health.

[53] Baumrind D (1971). Current patterns of parental authority. Dev Psychol.

[54] Ennett ST, Bauman KE, Foshee VA, Pemberton M, Hicks KA (2001). Parent–child Communication about Adolescent Tobacco and Alcohol Use: What Do Parents Say and Does It Affect Youth Behavior?. J Marriage Fam.

[55] Van Der Vorst H, Engels RCME, Meeus W, Deković M (2006). The impact of alcohol-specific rules, parental norms about early drinking and parental alcohol use on adolescents’ drinking behavior. J Child Psychol Psychiatry.

[56] Stanczyk N, Bolman C, Adrichem M, Candel M, Muris J, Vries H (2014). Comparison of Text and Video Computer-Tailored Interventions for Smoking Cessation: Randomized Controlled Trial. J Med Internet Res.

[57] Bandura A (2001). SOCIAL COGNITIVE THEORY: An Agentic Perspective. Annu Rev Psychol.

[58] Perry CL, Williams CL, Veblen-Mortenson S, Toomey TL, Komro KA, Anstine PS (1996). Project Northland: Outcomes of a Communitywide Alcohol Use Prevention Program during Early Adolescence. Am J Public Health.

[59] Komro KA, Perry CL, Williams CL, Stigler MH, Farbakhsh K, Veblen-Mortenson S (2001). How did Project Northland reduce alcohol use among adolescents? Analysis of mediating variables. Health Educ Res.

[60] Sniehotta FF, Schwarzer R, Scholz U, Schüz B (2005). Action planning and coping planning for long-term lifestyle change: theory and assessment. Eur J Soc Psychol.

[61] Van Osch L, Lechner L, Reubsaet A, Wigger S, De Vries H (2008). Relapse prevention in a national smoking cessation contest: Effects of coping planning. Br J Health Psychol.

[62] Miller WR, Rollnick S (2013). Motivational Interviewing: Helping People Change, 3rd Edition (Applications of Motivational Interviewing).

[63] Friederichs S, Bolman C, Oenema A, Guyaux J, Lechner L (2014). Motivational Interviewing in a Web-Based Physical Activity Intervention With an Avatar: Randomized Controlled Trial. J Med Internet Res.

[64] Friederichs SAH, Oenema A, Bolman C, Guyaux J, van Keulen HM, Lechner L (2013). Motivational interviewing in a web-based physical activity intervention: questions and reflections. Health Promotion International.

[65] Bickmore T, Giorgino T (2006). Health dialog systems for patients and consumers. J Biomed Inform.

[66] Del Hoyo-Barbolla E, Kukafka R, Arredondo MT, Ortega M (2006). A new perspective in the promotion of e-health. Stud Health Technol Inform.

[67] Connolly TM, Boyle EA, MacArthur E, Hainey T, Boyle JM (2012). A systematic literature review of empirical evidence on computer games and serious games. Comput Educ.

[68] DeSmet A, Van Ryckeghem D, Compernolle S, Baranowski T, Thompson D, Crombez G (2014). A meta-analysis of serious digital games for healthy lifestyle promotion. Prev Med.

[69] Tüzün H, Yılmaz-Soylu M, Karakuş T, İnal Y, Kızılkaya G (2009). The effects of computer games on primary school students’ achievement and motivation in geography learning. Comput Educ.

[70] Papastergiou M (2009). Digital Game-Based Learning in high school Computer Science education: Impact on educational effectiveness and student motivation. Comput Educ.

[71] Locke EA, Latham GP, Jr HFON, Drillings M (1994). Goal setting theory. Motivation: Theory and research.

[72] Crutzen R, Cyr D, Vries NK (2012). The Role of User Control in Adherence to and Knowledge Gained from a Website: Randomized Comparison Between a Tunneled Version and a Freedom-of-Choice Version. J Med Internet Res.

[73] De Vet E, Brug J, De Nooijer J, Dijkstra A, De Vries NK (2005). Determinants of forward stage transitions: a Delphi study. Health Educ Res.

[74] Schneider F, Osch Lv, Vries Hd: Identifying Factors for Optimal Development of Health-Related Websites: A Delphi Study Among Experts and Potential Future Users. J Med Internet Res 2012, `14(1). 10.2196/jmir.1863 PMC3374541 22357411

