# Supplementary material for: Web-based interventions to decrease alcohol use in adolescents: a Delphi study about increasing effectiveness and reducing drop-out
Source: BMC Public Health. 2015 Apr 9;15:340. doi: 10.1186/s12889-015-1639-z (PMC4404642; doi:10.1186/s12889-015-1639-z)
Supplement: Additional file 1: Table S1. — Effective parenting practices/styles/actions. Table S2. Social environmental factors. Table S3. Motivational factors. [file 12889_2015_1639_MOESM1_ESM.doc]

# Additional file 1

**Table S1**: *Effective parenting practices / styles / actions*

| **What are, according to your expertise, effective parenting practices / styles / actions to reduce binge drinking in 16-18 year old adolescents?** |
| --- |
| 1. Having expectations |
| 1. Communication    - About expectations not drink alcohol    - Firm    - Consistent    - Kind    - Open    - Healthy    - Positive quality |
| 1. Set (clear and consistent) rules |
| 1. Come to agreements |
| 1. Good role modeling (do not drink (much) in presence of child) |
| 1. Parental monitoring (knowing where and with who the child is)    - Know whereabouts    - Know friends |
| 1. Do not serve alcohol at home |
| 1. Responsive parenting |
| 1. Child management practices, care and control |
| 1. Family cohesion/bonding |
| 1. Family support / co-operation |
| 1. Active interest in adolescents life |
| 1. Doing activities that adolescent enjoys |
| 1. Authoritative warm and firm parenting |

**Table S2:** *Social environmental factors*

| **What are, according to your expertise, social environmental factors that determine binge drinking in adolescents aged 16-18 years (think of peers, parents, siblings etc.)?** |
| --- |
| 1. Parental approval |
| 1. Peers behavior |
| 1. Peers attitudes |
| 1. Parental attitudes |
| 1. Perceived normative drinking levels among age mates |
| 1. Older siblings providing alcohol to their younger siblings |
| 1. Availability (having alcohol at home) |
| 1. Peer pressure |
| 1. Parents function as role models (good or bad) |
| 1. Siblings behavior: older siblings willingness to use substances and their actual use are robust predictors of their young siblings later use |
| 1. Peer selection: selection of like-minded peers: mutual influence, interdependent |
| 1. Adolescents who are still non-users are more susceptible to the influence of their parents as models and sources of authority 2. Young people who enjoy a positive relationship with their parents may be less influenced by substance-using peers and less involved in alcohol using activities |
| 1. Family can continue to be a moderating influence throughout adolescence and even young adulthood |
| 1. Parents usually affect long term goals and values |
| 1. Parental drinking |
| 1. Activities of adolescent social group |
| 1. Role modeling in social environment is important (if youth see negative consequences occurring they may be hesitant to join in) |
| 1. Stress and coping styles puberty peer group |
| 1. Low image of drinking in moderation |
| 1. Parents who offer drinks |
| 1. Self-efficacy towards making agreements and setting rules |

**Table S3:** *Motivational factors*

| **What are, according to your expertise, motivational factors that determine binge drinking in adolescents aged 16-18 years? (Think of attitude, self-efficacy etc.)** |
| --- |
| 1. Acceptance in a peer group |
| 1. Importance of belonging to a group |
| 1. Insecurity in a group |
| 1. Looking for recognition in a group |
| 1. Boost self-confidence (children feel more confident through alcohol) |
| 1. Drinking to deal with negative emotions (drinking to cope) is related to alcohol-related consequences |
| 1. Drinking to enhance positive emotions (enhancement motives) |
| 1. Drinking to be social (social motives) are related to binge drinking |
| 1. Positive attitude towards binge drinking |
| 1. Low knowledge of consequences about the harm of binge drinking or negative consequences of alcohol |
| 1. Mental health |
| 1. Worse decision making skills |
| 1. Higher peer pressure susceptibility |
| 1. Negative attitude about school |
| 1. Prior school failure |
| 1. Positive drinking expectancies |
| 1. Normative expectations of peer drinking and adult drinking |
| 1. High self-efficacy over their ability to engage in binge drinking |
| 1. Low levels of perceived control over whether or not they could engage in binge drinking |
| 1. Low self-esteem |
| 1. Negative self-identity |
| 1. Excitement about trying out new things, curiosity |
| 1. Planned ahead of time |
| 1. Holidays and events encourage binge drinking |
| 1. Expectations that older teens and emerging adults drink at a “rite of passage” likely operates as well |
| 1. Personality  - Fearful - Sensation seeking - Impulsive |
| 1. Unable to point out personal boarders |
| 1. Building up brains has not finished |
| 1. They are short term thinkers |
| 1. Emphasize short term risks / benefits |
| 1. Positive attitudes towards substance use |
| 1. Intention to use |
| 1. Not sufficient self-efficacy and skills not to engage in these behaviors |
| 1. Quality of coping and social skills (including the skill to say no) are important because young people who set limits for themselves with regard to substance use which also have skill not to be persuaded by others, have a lower risk to develop problematic use such as binge drinking |
